# Relative frequencies and clinical features of Guillain-Barré Syndrome before and during the COVID-19 pandemic in North China

**DOI:** 10.1186/s12879-024-09401-1

**Published:** 2024-05-30

**Authors:** Yaqian Li, Rongjuan Zhao, Ling Li, Huiru Xue, Huaxing Meng, Guanxi Li, Feng Liang, Huiqiu Zhang, Jing Ma, Xiaomin Pang, Juan Wang, Xueli Chang, Junhong Guo, Wei Zhang

**Affiliations:** 1 Department of Neurology, First Hospital of Shanxi Medical University, Taiyuan, China; 2 First Clinical Medical College, Shanxi Medical University, Taiyuan, China

**Keywords:** Guillain-Barré Syndrome, COVID-19, Incidence, Prognosis, Characteristic

## Abstract

**Objective:**

Most studies investigated the relationship between COVID-19 and Guillain-Barré syndrome (GBS) by comparing the incidence of GBS before and during the pandemic of COVID-19. However, the findings were inconsistent, probably owing to varying degrees of the lockdown policy. The quarantine requirements and travel restrictions in China were lifted around December 7, 2022. This study aimed to explore whether the relative frequency of GBS increased during the major outbreak in the absence of COVID-19-mandated social restrictions in China.

**Methods:**

GBS patients admitted to the First Hospital, Shanxi Medical University, from December 7, 2022 to February 20, 2023, and from June, 2017 to August, 2019 were included. The relative frequencies of GBS in hospitalized patients during different periods were compared. The patients with and without SARS-CoV-2 infection within six weeks prior to GBS onset formed the COVID-GBS group and non-COVID-GBS group, respectively.

**Results:**

The relative frequency of GBS among hospitalized patients during the major outbreak of COVID-19 (13/14,408) was significantly higher than that before the COVID-19 epidemic (29/160,669, *P* < 0.001). More COVID-GBS patients (11/13) presented AIDP subtype than non-COVID-GBS cases (10/27, *P* = 0.003). The mean interval between onset of infective symptoms and GBS was longer in COVID-GBS (21.54 ± 11.56 days) than in non-COVID-GBS (5.76 ± 3.18 days, *P* < 0.001).

**Conclusions:**

COVID-19 significantly increased the incidence of GBS. Most COVID-GBS patients fell into the category of AIDP, responded well to IVIg, and had a favorable prognosis.

**Supplementary Information:**

The online version contains supplementary material available at 10.1186/s12879-024-09401-1.

## Introduction

Severe acute respiratory syndrome coronavirus 2 (SARS-CoV-2) emerged in late 2019 and has caused a pandemic of Coronavirus Disease 2019 (COVID-19) [1]. Several immune-mediated neurological disorders after COVID-19 infection have been reported, including Guillain-Barré syndrome (GBS) [2].

GBS is an important cause of acute flaccid paralysis and is characterized by symmetrical weakness of the limbs, with or without cranial and sensory nerve deficits, and hyporeflexia or areflexia [3]. The subtypes of GBS include acute inflammatory demyelinating polyneuropathy (AIDP), acute motor axonal neuropathy (AMAN), Miller Fisher syndrome (MFS), pharyngeal-cervical-brachial weakness (PCB), acute motor sensory axonal neuropathy (AMSAN) and paraparesis [4]. GBS typically occurs after an infectious disease in which an aberrant immune response damage peripheral nerves [4]. Molecular mimicry is proposed as a leading mechanism for the potential link between certain infections and the onset of GBS, where the immune response to pathogens could mistakenly target neural tissues due to similarities in protein structures [5]. Pathogens causing antecedent infections related to GBS contains *Campylobacter Jejuni*, cytomegalovirus, Zika virus, Epstein-Barr virus, *Mycoplasma pneumonia*, *Haemophilus influenzae*, Japanese encephalitis virus, influenza A virus, chikungunya virus, and dengue virus [4–6].

An association between COVID-19 and the subsequent development of GBS has been observed, with an increasing number of cases reported globally [7]. SARS-CoV-2 may trigger GBS through molecular mimicry, aligning with known infectious precipitants [8]. Most previous studies attempted to investigate the relationship between COVID-19 and GBS by comparing the incidence of GBS before and during the pandemic [9–11]. However, the findings were inconsistent probably owning to varying degrees of the lockdown policy [10, 11]. The association between COVID-19 and GBS should be explored in the absence of COVID-19-mandated social restrictions.

The sudden change of public health policy regarding COVID-19 in China around December 7, 2022 caused an immediate large-scale outbreak of infections across the entire country lasting for approximately 2 months. It was estimated that 80% people were infected in this major outbreak [12]. The period gave a good opportunity to figure out the relationship between COVID-19 and GBS. This study aimed to compare the relative frequencies of GBS during the major outbreak at the end of 2022 and before COVID-19 pandemic, and investigate the distinctive clinical characteristics and prognosis of GBS associated with COVID-19.

## Methods

### Study design and participants

**Fig. 1:**
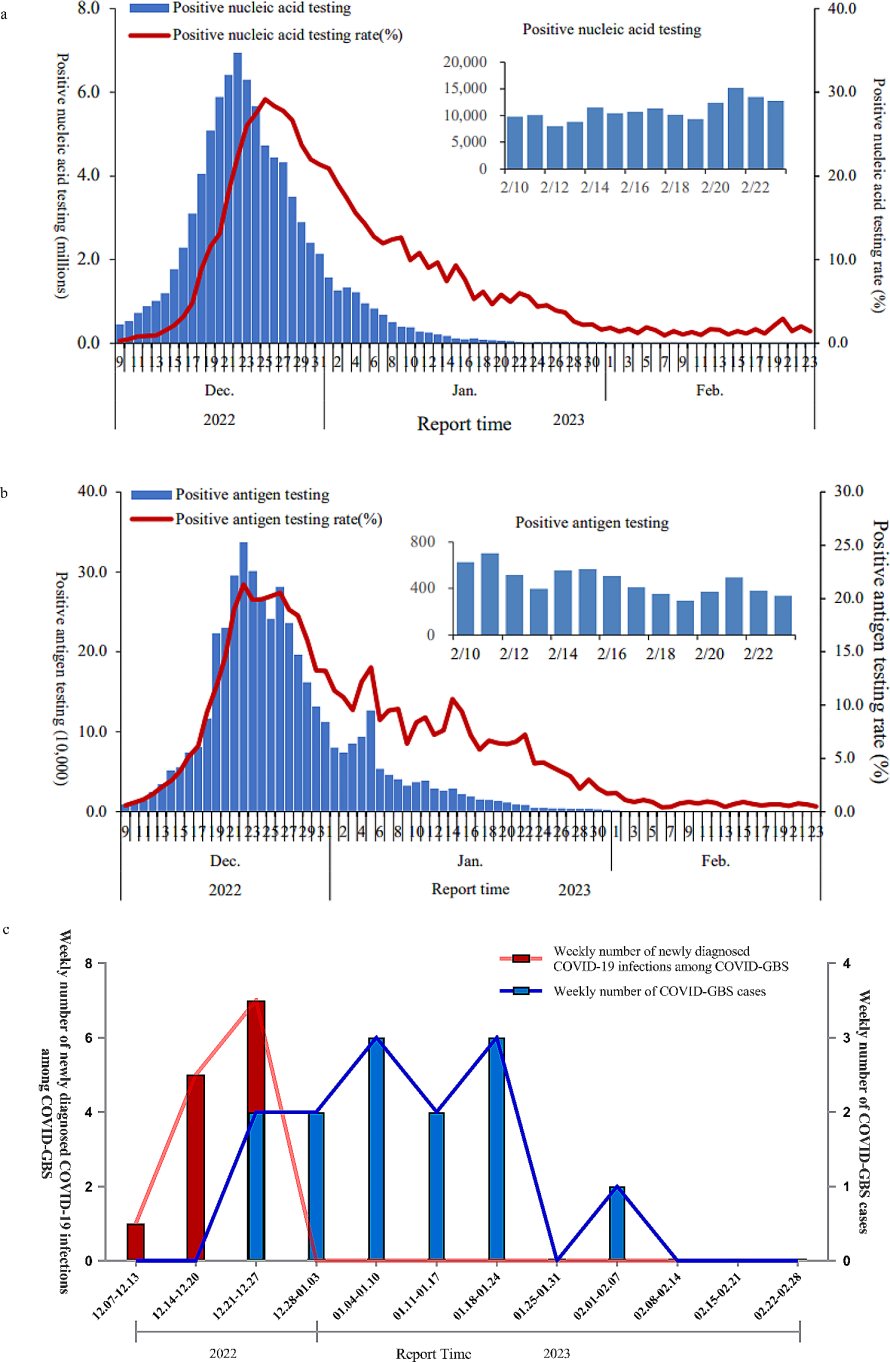
The daily number of reported Coronavirus Disease 2019 (COVID-19) cases and positive rate of COVID-19 testing in Chinese mainland (**a-b**) and the weekly number of COVID-Guillain-Barré syndrome (GBS) cases in our hospital (**c**) during the COVID-19 pandemic at the end of 2022. The COVID-19 was diagnosed based on SARS-Cov-2 RNA detection (**a**) or SARS-CoV-2 antigen (**b**)

The present study was a retrospective, observational, single-center study investigating the potential relationship between COVID-19 and GBS. The major outbreak began from December 7, 2022 and subsided around January 20, 2023 (Fig. 1a-b). All of the GBS patients admitted to the First Hospital, Shanxi Medical University, from December 7, 2022 to February 20, 2023, and from June, 2017 to August, 2019 were included. The diagnosis of GBS was established using a previous criterion [13]. The relative frequencies of GBS before (from June, 2017 to August, 2019) and during the major outbreak (from December 7, 2022 to February 20, 2023) were compared. As cold season GBS peak has been reported [14], we also calculated the relative frequencies of GBS before and during the major outbreak within the same period across different years.

The patients with and without SARS-CoV-2 infection within six weeks prior to GBS onset formed the COVID-GBS group and non-COVID-GBS group, respectively. Another 110 COVID-19 hospitalized patients without GBS during the major outbreak were randomly selected as COVID-non-GBS group. None of these 110 patients had clinical symptoms or a diagnosis of GBS during hospitalization.

Information was collected from medical records, including demographic characteristics, related pre-illness conditions, clinical symptoms and signs, severity, Medical Research Council (MRC)-sumscores at nadir and discharge [15], laboratory and electrophysiological data, and treatment. The prognosis of COVID-GBS was investigated by telephone interview within three months after onset with GBS disability scale [16]. The clinical characteristics were compared between COVID-GBS and non-COVID-GBS and between COVID-GBS and COVID-non-GBS.

The diagnosis of GBS was made by a consensus of two neurologists in the Department of Neurology at our center on the basis of clinical presentation, cerebrospinal fluid (CSF) analysis, and electrophysiological studies recorded during hospitalization. The diagnoses of AMAN and AIDP were confirmed using Hadden’s electrodiagnostic criteria [17]. AMSAN was diagnosed by an absence of demyelinating features, as in Hadden’s criteria, and reduction in sensory nerve action potential amplitude < 50% of the lower limit of normal in at least two nerves [18, 19]. The COVID-19 was diagnosed based on SARS-Cov-2 RNA detection by reverse transcriptase polymerase chain reaction (RT-PCR) or SARS-CoV-2 antigen. The severity of COVID-19 was classified into asymptomatic or mild, moderate, severe and critical types according to the China COVID-19 guideline [20].

### Statistics

Continuous data were expressed as means ± standard deviation (SD) or medians (range), and discrete variables were expressed as absolute values and percentages. Relative frequencies of GBS in patients hospitalized were calculated and compared between different periods with Poisson test. Percentages were compared between groups using Fisher exact test. Student t-test or Mann-Whitney U test was used to compare continuous variables depending on the distribution types. Two-tailed *P*-values < 0.05 were considered statistically significant. Descriptive statistics and preliminary data processing were performed using SPSS version 25.0 (IBM Corp, Armonk, New York). Advanced analyses, including the Poisson test, were conducted with R statistical software (version 4.2.3, The R Foundation for Statistical Computing). GraphPad Prism version 9.0 was used for the graphical representation.

## Results

### Epidemiological dynamics of the outbreak

According to the report from Chinese Center for Disease Control and Prevention (CCDC) [21], the outbreak of COVID-19 at the end of 2022 occurred between December 2022 and January 2023 (Fig. 1a-b). In total, 13 cases of GBS were diagnosed around the period, all of which had suffered from COVID-19 within six weeks prior to GBS onset. The first case developed symptoms of GBS at December 21, 2022 on week 3 of the outbreak, while the peaks of the COVID-19 epidemic and GBS cases were reached on week 3 and 5, respectively (Fig. 1c).

The relative frequency of GBS among all patients hospitalized in our hospital between December 7, 2022 and February 20, 2023 was 13/14,408 (0.090%). There were 29 cases with GBS identified between June, 2017 and August, 2019 in our hospital, with a relative frequency of 29/160,669 (0.018%). The relative frequency of GBS during the major outbreak of COVID-19 was significantly higher than that before the COVID-19 epidemic (Poisson test, RR 5.00, 95%CI 2.39–9.92, *P* < 0.001, Table 1). To exclude the seasonal effect, the relative frequency of GBS between December 7, 2017 and February 20, 2018 was calculated (3/12,513, 0.024%), also revealing a higher frequency during the major outbreak (Poisson test, RR 3.76, 95%CI 1.03–20.59, *P* = 0.041).

**Table 1:** The demographic and clinical characteristics between COVID-GBS and non-COVID-GBS

	COVID-GBS		Non-COVID-GBS	*P* value
n (%)		13	29	
Relative frequencies, %		13/14,408 (0.090)	29/160,669 (0.018)	< 0.001
Male, n (%)		12 (92.3)	21 (72.4)	0.232
Age (mean ± SD)		53.92 ± 18.24	45.38 ± 16.40	0.139
GBS subtypes, n (%)				0.003
AIDP		11 (84.6)	10/27 (37.0)	0.005
AMAN		1 (7.7)	15/27 (55.6)	0.005
AMSAN		0	2/27 (7.4)	1.000
Miller-Fisher		0	0	NA
Undetermined		1(7.7)	0	0.325
Preceding infections, n (%)		13 (100)	20 (68.9)	0.038
Clinical respiratory tract infection		13 (100)	9 (31.0)	0.000
Clinical gastroenteritis/diarrhea		0 (0)	11 (37.9)	0.009
No symptoms of infection		0 (0)	9 (31.0)	0.038
Days from infection to GBS onset, mean ± SD		21.54 ± 11.56	5.76 ± 3.18	0.000
Signs and symptoms, n (%)				
Limb weakness		13 (100)	29 (100)	NA
Cranial nerve involvement		10 (76.9)	11 (37.9)	0.019
Sensory loss		9 (69.2)	12/28 (42.9)	0.116
Paresthesia		9 (69.2)	13/28 (46.4)	0.173
Days from GBS onset to nadir, median (range)		7 (2–28)	10 (3–34)	0.230
Severity at nadir, n (%)				
Unable to walk unaided		8 (61.5)	19 (65.5)	1.000
Endotracheal intubation		1 (7.7)	4 (13.8)	0.961
Gastric intubation		1 (7.7)	4 (13.8)	0.961
MRC-sumscores at nadir, median (range)		45 (12–54)	44 (12–56)	0.558
GBS disability score at nadir, median (range)		4 (2–5)	NA	
Lumbar puncture, n (%)		10 (76.9)	23 (79.3)	
CSF protein concentrations, median (range)		1.735 (0.27–4.42)	0.51 (0.32–1.9)	0.021
CSF albuminocytological dissociation^*^, n (%)		8/10 (80)	15/23 (65.2)	0.682
Serum IgM or IgG reactivity against glycolipid, n (%)		2/9 (22.2)	3/7 (42.9)	0.596
Therapy, n (%)				0.528
IVIg		12 (92.3)	28 (96.6)	
None		1 (7.7)	1 (3.4)	
Hospital stays, median (range)		10 (5–46)	9 (5–56)	0.469
MRC-sumscores at discharge, median (range)		52 (24–60)	51 (18–60)	0.257
MRC-sumscore changes from nadir to discharge, median (range)		2 (0–32)	6 (0–38)	0.360
Short-term prognosis				
Follow-up interval, days, median (range)		34 (20–76)	NA	
GBS disability score, median (range)		2 (0–3)	NA	

Abbreviations: GBS = Guillain-Barré syndrome; AMAN = acute motor axonal neuropathy; AIDP = acute inflammatory demyelinating polyneuropathy; AMSAN = acute motor sensory axonal neuropathy; NA = not applicable; MRC = Medical Research Council; CSF = cerebrospinal fluid; IVIg = intravenous immunoglobulin

^*^ cell count < 50 cell/µl with elevated CSF proteins

### Clinical characteristics of COVID-GBS and non-COVID-GBS

The demographics and clinical characteristics of patients with COVID-GBS and non-COVID-GBS are shown in Table 1. The mean age of patients with COVID-GBS was 53.92 ± 18.24 years and most were male (12/13). The age and sex differences between the two groups were comparable (*P* > 0.05). AIDP was the chief subtype in patients with COVID-GBS (11/13), and AMAN was more prevalent in non-COVID-GBS (15/27, *P* = 0.003). Correspondingly, more non-COVID-GBS patients (11/29) reported preceding gastrointestinal symptoms than COVID-GBS patients (0/13, *P* = 0.009). The mean interval between onset of infective symptoms and GBS was longer in COVID-GBS (21.54 ± 11.56 days) than in non-COVID-GBS (5.76 ± 3.18 days, *P* < 0.001). All but one case with COVID-19 developed GBS while the symptoms of COVID-19 going away. Cranial nerve involvement was more common in COVID-GBS (10/13) than in non-COVID-GBS (11/29, *P* = 0.019). The intervals between GBS onset and nadir and MRC-sumscores at nadir were similar between the two groups (*P* > 0.05).

CSF albuminocytologic dissociation (cell count < 50 cell/µl with elevated CSF proteins) [22] was noted in more than two-thirds of patients in both groups (*P* = 0.0682). The protein levels in CSF were significantly higher in COVID-GBS than in non-COVID-GBS (*P* = 0.021). Antiganglioside antibodies were uncommonly detected (2/9) in COVID-GBS, but the difference between the two groups was not significant (*P* = 0.596).

Most patients in both groups were treated with intravenous immunoglobulin (IVIg). The lengths of hospital stays were similar between the two groups (*P* = 0.469), and the severity at nadir and discharge was comparable between the two groups as reflected by MRC-sumscores (*P* = 0.257). Most patients with COVID-GBS responded well to IVIg and they achieved a median GBS disability score of 2 (able to walk 10 m or more without assistance but unable to run) within 3 months from a median score of 4 at nadir.

### Clinical characteristics of COVID-19 in patients with GBS and non-GBS

The clinical characteristics of patients with COVID-GBS and COVID-non-GBS are shown in Table 2. Compared with COVID-GBS group (51 [27–85]), COVID-non-GBS patients were older (73[14–94], *P* < 0.002). Patients with COVID-non-GBS had a more-balanced gender ratio with male accounting for 56.4% (*P* < 0.012). Patients in the COVID-non-GBS group (3 [1-26]) had longer durations of COVID-19 respiratory symptoms before admission than those in the COVID-GBS group (7 [0–30], *P* = 0.033). The lengths of hospital stays were similar between the two groups (*P* = 0.382). COVID-non-GBS patients had more severe symptoms of COVID-19 than COVID-GBS patients. More than two-thirds of COVID-GBS patients (9/13, 69.2%) were asymptomatic or mild types of COVID-19, while only 21.8% in COVID-non-GBS group fell into the corresponding types (9/13 vs. 24/110, *P* = 0.001).

**Table 2:** The demographic and clinical characteristics between COVID-GBS and COVID-non-GBS

	COVID-GBS	COVID-non-GBS	*P* value
n, %	13	110	
Age, median (range)	51 (27–85)	73 (14–94)	0.002
Male, n (%)	12 (92.3)	62 (56.4)	0.012
Hospital stays, median (range)	10 (5–46)	10.5 (1–63)	0.382
Durations of COVID-19 respiratory symptoms before admission, median (range) / n	3 (1–26) / 13	7 (0–30) / 109	0.033
COVID-19 types, n (%)			0.008
Asymptomatic or mild type	9 (69.2)	24 (21.8)	0.001
Moderate type	2 (15.4)	37 (33.6)	0.223
Severe type	1 (7.7)	31 (28.2)	0.180
Critical type	1 (7.7)	18 (16.4)	0.692
Signs of COVID-19, n (%)			
Fever	10 (76.9)	77 (70.0)	0.754
Cough	6 (46.2)	85 (77.2)	0.039
Dyspnea	2 (15.4)	62 (56.4)	0.007
Endotracheal intubation	1 (7.7)	13 (11.8)	1.000
Olfactory and gustatory disorders	1/12 (8.3)	1/7 (14.3)	1.000
Signs at hospital arrival			
Temperature (℃), mean ± SD	36.4 ± 0.22	36.69 ± 0.61	0.140
Heart rate (bpm), mean ± SD	76.9 ± 4.47	85.2 ± 15.71	0.000
Respiratory rate (bpm), mean ± SD	19.5 ± 1.20	20.2 ± 2.88	0.360
Laboratory findings at nadir			
White blood cell count (*10^9/L), median (range) / n	8.6 (3.1–19.0) / 13	8.7 (2.2–49.3) / 109	0.472
Elevated white blood cell count, n (%)	5/11 (45.5)	51/109 (46.8)	1.000
Decreased white blood cell count, n (%)	1/11 (9.1)	16/109 (14.7)	1.000
Hemoglobin count (g/L), mean ± SD / n	134.9 ± 22.60 / 11	112.5 ± 23.64 / 109	0.003
Blood platelet count (*10^9/L), median (range) / n	281 (128–481) / 11	192 (17–555) / 109	0.019
Elevated C-reactive protein, n (%)	4/9 (44.4)	62/75 (82.7)	0.019
AST (U/L), median (range) / n	28 (19–104) / 12	35.5 (7-534) / 108	0.428
ALT (U/L), median (range) / n	27 (11–157) / 12	35 (5-323) / 108	0.564
Creatinine (mg/dl), median (range) / n	54.7 (24.5–86) / 12	67.1 (39.3–683) / 106	0.033
Serum potassium (mmol/L), median (range) / n	3.70 (3.00-4.12) / 12	3.80 (2.16–5.09) / 106	0.634
Serum sodium (mmol/L), median (range) / n	135.5 (111–146) / 12	138.0 (110–156) / 105	0.407
Serum chlorine (mmol/L), median (range) / n	101.0 (81.3-107.2) / 12	101.5 (74.9–116.0) / 106	0.431

Abbreviations: AST = aspartate aminotransferase; ALT = alanine aminotransferase

## Discussion

In this study, we found that there was a sudden increase in the number of GBS patients during the peak pandemic months, indicating an association between COVID-19 and GBS. Previous studies investigated the relationship between COVID-19 and GBS mainly by comparing the incidence of GBS before and during the pandemic [9–11]. The majority of the studies resulted in negative outcomes [9–11]. In fact, other factors in addition to COVID-19 affected the incidence of GBS. Strict lockdown policy reduced the number of GBS patients [10, 11]. A low prevalence of COVID-19 might diminish its effect on GBS [10]. During the major outbreak in China starting from December 7, 2022, 80% people were infected in the absence of social restrictions. Under this background, the relative frequency of GBS in hospitalized patients in the same hospital during the major outbreak in China was 5-fold higher than that before the epidemic of COVID-19 in the present study, which provided additional evidence for that COVID-19 was associated with GBS. Case-control studies are commonly used to look at factors associated with diseases [23]. One case-control study conducted in Spain revealed that SARS-CoV-2 infection was associated with a 6.3-fold increase in the incidence of GBS [24]. Another nested case-control study also suggested that SARS-CoV-2 infection was associated with increased risk of GBS [25].

The preceding infections influence the subtypes of GBS. C. *jejuni* and Zika virus infections are strongly associated with AMAN [26]. Most patients with GBS after influenza virus infection are categorized into AIDP [27]. AMAN was the most prevalent type of GBS in China and in our region [28, 29]. However, during the major outbreak, nearly all of the cases (11/13) with GBS were classified as AIDP, which was consistent with previous studies [7, 30], providing phenotypic evidence of association between SARS-CoV-2 infection and GBS. Accordingly, COVID-GBS patients exhibited higher prevalence of cranial nerve involvement and CSF protein concentrations, similar to patients with AIDP [4, 31].

The symptoms of COVID-19 in most patients with COVID-GBS were mild in this study, different from previous case reports [7], which indicated that occurrence of GBS was independent of the severity of COVID-19. At the time of GBS onset, the symptoms of COVID-19 disappeared in most COVID-GBS patients, suggesting a course of immune-mediated disease rather than virus damaging directly.

Although COVID-19 increases the incidence of GBS, it is not known whether the immune response toward SARS-CoV-2 damage peripheral nerves. An alternative hypothesis is that there is a secondary infection due to immune suppression caused by COVID-19, which induces GBS [32, 33]. Zhu et al. revealed that 242 of 257 COVID-19 patients (94.2%) were co-infected with one or more respiratory pathogens, several of which could cause GBS, including *Haemophilus influenzae*, Epstein-Barr virus, influenza A virus, *Mycoplasma pneumonia*, and cytomegalovirus [34]. In the present study, the COVID-GBS patients had a significantly longer average interval (21.54 ± 11.6 days) between preceding infections and GBS than those with non-COVID-GBS (5.76 ± 3.18 days), which supported the hypothesis. A systemic review included 436 COVID-GBS patients also indicated a similar mean interval (19 days) [35]. On the contrary, for example, the median time between infectious to neurologic symptoms was 3, 5 and 6 days in GBS patients associated with C. *jejuni*, Japanese encephalitis virus, and Zika virus infection, respectively [36–38]. An epidemiological study conducted in Finland revealed a mean interval of 12.4 days [39]. It is reasonable to speculate that a secondary infection following COVID-19 causes GBS, and therefore the interval between SARS-CoV-2 infection and GBS gets longer. Further evidence was needed to elucidate the molecular mechanism why COVID-19 increases the incidence of GBS [40].

## Limitations

There are several limitations to our study. First, the study has a small sample size, which increases the sampling error. Meanwhile, this retrospective pilot study may have omitted some key information, and the completeness of the original data cannot be assured due to the nature of data collection post-case identification. Second, we recruited patients from a single hospital and the precise number of COVID-19 patients in the region was not available, and thus the incidence of GBS in COVID-19 patients could only be approximatively calculated and compared. Third, while our study suggests a potential link between secondary infections and the development of COVID-GBS, we did not specifically test for the immunoreactivity of the most prevalent infections in our geographic region among the patients. This limitation means that our conclusions are based on clinical inference rather than direct immunological evidence, which could provide a more definitive causal relationship.

## Conclusions

COVID-19 significantly increased the incidence of GBS. Most COVID-GBS patients fell into the category of AIDP, responded well to IVIg, and had a favorable prognosis. Further studies investigating the co-infected pathogens among COVID-GBS patients and focusing on the molecular mechanisms of the association between COVID-19 and GBS should be conducted.

### Electronic supplementary material

Below is the link to the electronic supplementary material.

Supplementary Material 1

Supplementary Material 2

Supplementary Material 3

Supplementary Material 4

Supplementary Material 5

Supplementary Material 6

## Data Availability

All data generated or analysed during this study are included in this published article and its supplementary information files.
