# Supplementary material for: Relative frequencies and clinical features of Guillain-Barré Syndrome before and during the COVID-19 pandemic in North China
Source: BMC Infect Dis. 2024 May 30;24:541. doi: 10.1186/s12879-024-09401-1 (PMC11138026; doi:10.1186/s12879-024-09401-1)
Supplement: Supplementary file 1 — Supplementary Material 1 [file 12879_2024_9401_MOESM1_ESM.pdf]

临床研究（科研） - 伦理审查批件

批件号：NO.KYLL-2023-133

项目名称	新型冠状病毒感染相关吉兰-巴雷综合征临床特征分析		
研究类别	☐已立项科研　☑拟开展科研　☐研究生培养		
科室/专业名称	神经内科/神经病学	主要研究者	张炜
审查时间	2023 -05- 11	地点	护校耳鼻喉实验室一层会议室
批准材料	☑伦理初始审查申请表；<br>☐ 伦理复审申请表；<br>☐ 伦理修正案申请表；<br>☑临床研究方案（版本号 V1.0，版本日期 2023-02-02）；<br>☐ 知情同意书；<br>☑ 主要研究者履历与 GCP/伦理培训证书；<br>☐ 科研课题立项文件；<br>☐ 招募材料；<br>☐ 临床研究原始记录（版本号 V1.0，日期 20 - - ）；<br>☑ 其他：免除签署知情同意书（知情同意例外申请、临床研究保密承诺）	研究单位	山西医科大学第一医院
审查情况	**初始审查方式**<br>☑快速审查<br>☐会议审查<br>会议时间：<br>（审查意见另附会议签到副本）<br>**复审方式**<br>☑快速审查<br>☐会议审查<br>会议时间：<br>（审查意见另附会议签到副本）	投票结果	☐初审结论：同意<br>☑复审结论：同意

年度/定期跟踪审查频率	12 个月，<br>➢ 请于 2023 年 5 月 18 日 前 1 个月提交年度/定期跟踪审查（研究进展报告）
批件有效期	2023 年 5 月 18 日 ~ 2024 年 5 月 18 日<br>➢ 若在批件有效期内研究项目没有启动，请重新提交伦理初始审查。<br>➢ 在研项目：若超出批件有效期，没有递交《研究进展报告》及获得伦理审查批准继续研究，研究者必须立即停止所有研究活动，包括干预措施和数据收集。假若停止研究干预可能会对受试者造成伤害，研究者应当要求伦理委员会批准在研的受试者继续参与研究。
注意事项	根据国家卫健委《涉及人的生物医学研究伦理审查办法》（2016）、国家药品监督管理局、国家卫健委《医疗器械临床试验质量管理规范》（2022）、国家卫健委《医疗卫生机构开展研究者发起的临床研究（试行）》（2021）的伦理原则，经本伦理委员会审查，同意开展该临床研究。<br>请研究团队开展研究之前做好方案、知情同意、受试者保护等培训，并保留培训记录备查。<br>凡涉及中国人类遗传资源、需要报批的研究项目，应获得中国人类遗传资源管理办公室批准后才能开始研究。<br>对于跨国/境研究的开展，应充分考虑并遵循研究所在国/境的法律、法规、政策和指南，以及当地的社会文化特点，做好受试者保护工作。<br>完成临床研究，请提交研究完成报告/结题报告。若发生严重不良事件以及影响研究风险与受益比的非预期不良事件，或违反方案等情况应及时报告本伦理委员会；暂停/终止研究应报告本伦理委员会。<br>若临床研究方案、知情同意书有修改，或主要研究者更换，应及时通知伦理委员会，重新审查，获得批准后执行。
联系方式（委员/秘书）	科研伦理秘书：智陞雯　　　　联系电话：　0351-4639021
主任委员/被授权者签名	[signature]<br>日期：2023 年 5 月 18 日<br>山西医科大学第一医院伦理委员会

申明：本伦理委员会严格按照中国 GCP 及相关法规组成和工作。
