# Supplementary material for: Relative frequencies and clinical features of Guillain-Barré Syndrome before and during the COVID-19 pandemic in North China
Source: BMC Infect Dis. 2024 May 30;24:541. doi: 10.1186/s12879-024-09401-1 (PMC11138026; doi:10.1186/s12879-024-09401-1)

**Clinical Research（Scientific Research）- Ethical review approval**

**Approval Number：NO. KYLL-2023-133**

| Project Name | Guillain-Barré Syndrome outbreak caused by COVID-19 at the end of 2022 in China | | | | |
| --- | --- | --- | --- | --- | --- |
| Research Category | □Ongoing research project ☑To be carried out research project □Postgraduate student training | | | | |
| Clinical Department/  Specialty | Neurology | Main Researchers | Zhang Wei | | |
| Review Time | 2023-05-11 | Review Location | Conference Room of the First Hospital of Shanxi Medical University | | |
| Approved Materials | ☑Application form for the initial ethics review;  ☑Application form for following ethics review;  □Application form for ethics amendment;  ☑Clinical study protocol;  □Informed consent;  ☑The main researchers’ resume and GCP/ Ethical training certificate;  □Scientific research project approval document;  □Recruit materials;  □Original records of the clinical studies;  ☑Others: Application for exceptions to informed consent, Commitment to clinical research confidentiality; | | | Research Organization | The First Hospital of Shanxi Medical University |
| Review status | Initial review method  ☑Quick review;  □Meeting review;  Following review method  ☑Quick review;  □Meeting review; | | | Poll  Result | □Initial review conclusion: approved  ☑Following review conclusion: approved |
| Following Review Frequency | 12 months. | | | | |
| Validity of Approval | May 18, 2023 - May 18, 2024  If the research project is not started within the validity period of the approval, please resubmit the initial ethics review.  For ongoing research projects: If the validity period of the approval is exceeded, and the “Research progress report” is not submitted and approved by ethic review, the investigator must immediately stop all study activities, including intervention measures and data collection. If discontinuation of the research intervention may cause harm to the subjects, the investigator should ask the Ethic Committee to approve the subject’s continued participation in the study. | | | | |
| Precautions | According to the National Health and Medical Commissions’ “Measures for the Ethical Review of Biomedical Research Involving Humans”’(2016), the National Drug Administration, the National Health and Medical Commission’s “Medical Device Clinical Trial Quality Management and Regulations”(2022), the National Health and Medical Commission’s “Medical and Health Instructions Carrying out Investigator- Initiated Clinical Research (Trial Implementation)”(2021) Ethical principles, the clinical research is agreed to be carried out after reviewed by the Ethic Committee.  The research team is requested to prepare the training on protocols, informed consent, and subject protection before carrying out the research, and keep training records for future reference.  Any research project involving Chinese human genetic resources that needs to be submitted for approval shall be approved by the China Human Genetic Resources Management Office before the research can begin.  For the development of cross-border research, the laws, regulations, policies and guidelines of the country / border where the research institute is located should be fully considered and followed, as well as the local social and cultural characteristics, and the subject protection work should be done well.  To complete the clinical research please submit the research completion report / conclusion report. In case of serious adverse events, unexpected adverse events affecting the research risk- benefit ratio, or violation of the protocol, etc., the ethics committee should be reported in time; the suspension / termination of the research should be reported to the ethics committee.  If the clinical research protocol or informed consent form is modified, or the main investigator is replaced, the ethics committee should be notified in time, re-reviewed, and implemented after approval. | | | | |
| Contact Information  (committee/secretary) | Research Ethics Secretary: Shengwen Zhi, (0086) 0351- 4639021 | | | | |
| Chairman/ Authorized person’s signature | 2023-05-18 | | | | |

Statement: The Ethic Committee is formed and works in strict accordance with China’s GCP and relevant laws and regulations.

Medical Ethics Committee of the First Hospital of Shanxi Medical University


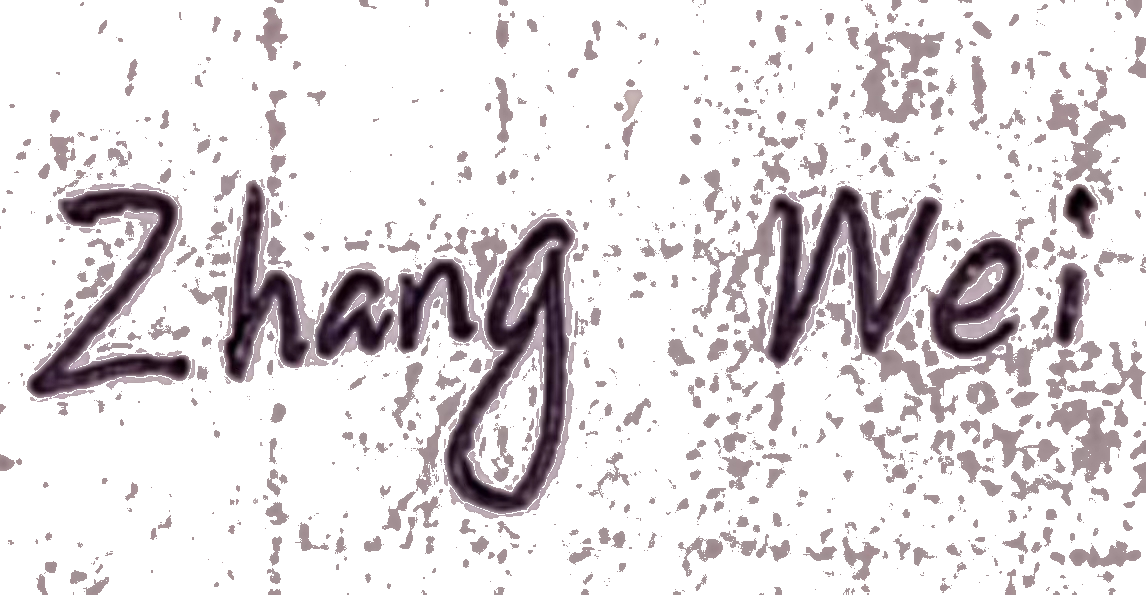

Supplement: Supplementary file 5 — Supplementary Material 5 [file 12879_2024_9401_MOESM5_ESM.doc]
