# Supplementary material for: Relative frequencies and clinical features of Guillain-Barré Syndrome before and during the COVID-19 pandemic in North China
Source: BMC Infect Dis. 2024 May 30;24:541. doi: 10.1186/s12879-024-09401-1 (PMC11138026; doi:10.1186/s12879-024-09401-1)
Supplement: Supplementary file 6 — Supplementary Material 6 [file 12879_2024_9401_MOESM6_ESM.docx]

**Supplementary Table S1** The demographic and clinical records of COVID-GBS patients.

| Patient | 1 | 2 | 3 | 4 | 5 | 6 |
| --- | --- | --- | --- | --- | --- | --- |
| GBS subtypes | AIDP | AIDP | AIDP | AIDP | AIDP | AIDP |
| Admission date | 2023/2/10 | 2023/2/20 | 2023/2/4 | 2023/2/3 | 2023/1/11 | 2022/12/30 |
| Date of onset | 2023/2/3 | 2023/1/15 | 2023/1/20 | 2023/1/22 | 2023/1/4 | 2022/12/26 |
| Date of confirmed COVID-19 infection | 2022/12/24 | 2022/12/20 | 2022/12/21 | 2022/12/24 | 2022/12/20 | 2022/12/23 |
| Days from infection to GBS onset | 41 | 27 | 30 | 30 | 15 | 3 |
| Signs and symptoms |  |  |  |  |  |  |
| Limb weakness | Yes | Yes | Yes | Yes | Yes | Yes |
| Cranial nerve involvement | No | Yes | Yes | Yes | Yes | Yes |
| Sensory loss | No | Yes | No | Yes | Yes | Yes |
| Paresthesia | Yes | Yes | No | Yes | Yes | No |
| Days from GBS onset to nadir | 7 | 28 | 12 | 12 | 7 | 2 |
| Severity at nadir |  |  |  |  |  |  |
| Unable to walk unaided | No | Yes | Yes | Yes | Yes | Yes |
| Endotracheal intubation | No | No | No | No | No | No |
| Gastric intubation | No | No | No | No | No | No |
| MRC-sumscores at nadir | 54 | 45 | 12 | 54 | 36 | 46 |
| GBS disability score at nadir | 2 | 4 | 4 | 3 | 4 | 3 |
| CSF protein concentrations, g/L | NA | 3.40 | NA | 1.04 | 2.34 | 0.27 |
| CSF albuminocytological dissociation | NA | Yes | NA | Yes | Yes | No |
| Types of ganglioside antibodies | NA | serum GM2-IgM (++), GD2-IgM (+) | (-) | (-) | (-) | (-) |
| Therapy | IVIg | IVIg | IVIg | IVIg | IVIg | IVIg |
| Hospital stays | 5 | 8 | 10 | 11 | 10 | 6 |
| MRC-sumscores at discharge | 56 | 45 | 24 | 56 | 36 | 52 |
| MRC-sumscore changes from nadir to discharge | 2 | 0 | 12 | 2 | 0 | 6 |
| Short-term prognosis, GBS disability score | 1 | 3 | 3 | 2 | 2 | 2 |

**Supplementary Table S1 (continued)** The demographic and clinical records of COVID-GBS patients.

| Patient | 7 | 8 | 9 | 10 | 11 | 12 | 13 |
| --- | --- | --- | --- | --- | --- | --- | --- |
| GBS subtypes | AIDP | AIDP | AMAN | AIDP | AIDP | Undetermined | AIDP |
| Admission date | 2023/1/5 | 2023/1/19 | 2023/2/8 | 2023/1/29 | 2022/12/28 | 2023/1/6 | 2023/2/8 |
| Date of onset | 2022/12/30 | 2023/1/10 | 2023/1/14 | 2023/1/10 | 2022/12/21 | 2022/12/31 | 2023/1/18 |
| Date of confirmed COVID-19 infection | 2022/12/24 | 2022/12/15 | 2022/12/17 | 2022/12/14 | 2022/12/11 | 2022/12/22 | 2022/12/23 |
| Days from infection to GBS onset | 6 | 26 | 28 | 28 | 10 | 9 | 27 |
| Signs and symptoms |  |  |  |  |  |  |  |
| Limb weakness | Yes | Yes | Yes | Yes | Yes | Yes | Yes |
| Cranial nerve involvement | Yes | No | No | Yes | Yes | Yes | Yes |
| Sensory loss | Yes | Yes | No | No | Yes | Yes | Yes |
| Paresthesia | No | Yes | Yes | No | Yes | Yes | Yes |
| Days from GBS onset to nadir | 7 | 5 | 3 | 7 | 5 | 6 | 21 |
| Severity at nadir |  |  |  |  |  |  |  |
| Unable to walk unaided | No | Yes | Yes | No | No | Yes | No |
| Endotracheal intubation | Yes | No | No | No | No | No | No |
| Gastric intubation | Yes | No | No | No | No | No | No |
| MRC-sumscores at nadir | 26 | 18 | 50 | 54 | 52 | 45 | 42 |
| GBS disability score at nadir | 5 | 4 | 4 | 3 | 3 | 3 | 4 |
| CSF protein concentrations, g/L | 1.30 | 4.42 | 0.36 | NA | 2.97 | 0.89 | 2.17 |
| CSF albuminocytological dissociation^*^ | Yes | Yes | No | NA | Yes | Yes | Yes |
| Types of ganglioside antibodies | NA | (-) | NA | (-) | serum GD1a-IgG (+) | (-) | NA |
| Therapy | IVIg | IVIg | IVIg | IVIg | IVIg | None | IVIg |
| Hospital stays | 46 | 10 | 12 | 5 | 6 | 11 | 6 |
| MRC-sumscores at discharge | 58 | 48 | 50 | 54 | 52 | 60 | 54 |
| MRC-sumscore changes from nadir to discharge | 32 | 30 | 0 | 0 | 0 | 15 | 12 |
| Short-term prognosis, GBS disability score | 2 | 2 | 2 | 2 | 0 | 0 | 2 |

Abbreviations: GBS = Guillain-Barré syndrome; AMAN = acute motor axonal neuropathy; AIDP = acute inflammatory demyelinating polyneuropathy; NA = not available; MRC = Medical Research Council; CSF = cerebrospinal fluid; IVIg = intravenous immunoglobulin.

^*^ cell count < 50 cell/μl with elevated CSF proteins

**Supplementary Table S2** The electrophysiological records of COVID-GBS patients.

| Patient | 1 | 2 | 3 | 4 | 5 | 6 |
| --- | --- | --- | --- | --- | --- | --- |
| **Motor nerve conduction** |  |  |  |  |  |  |
| **Median nerve** |  |  |  |  |  |  |
| Distal latency (ms) | 5.41/5.17 | 5.88/NR | 10.1/10.9 | 19.9/27.7 | 3.3/3.93 | 3.96/3.73 |
| CMAP amplitude (mv) | 6.2/7.1 | 1.01/NR | 2.7/1.32 | 0.93/0.32 | 9.6/9.7 | 5.0/6.4 |
| Conduction velocity (m/s) | 45.0/49.9 | 38.9/NR | 34.9/31.9 | 30.0/39.3 | 54.1/54.7 | 53.3/38.0 |
| F wave latency (ms) | 28.0/27.1 | NR/NR | NR/NR | NR/NR | 31.0/30.5 | 32.7/NR |
| Conduction block | No | No | No | No | No | Yes |
| Temporal dispersion | No | No | Yes | Yes | No | No |
| **Ulnar nerve** |  |  |  |  |  |  |
| Distal latency (ms) | 3.59/3.46 | 5.32/4.02 | 5.69/5.19 | 7.88/5.41 | 2.99/3.15 | 2.99/2.5 |
| CMAP amplitude (mv) | 3.3/3.8 | 1.96/0.96 | 3.2/2.9 | 1.3/0.99 | 7.4/8.1 | 7.4/8.6 |
| Conduction velocity (m/s) | 57.8/57.1 | 45.1/40.2 | 46.2/38.6 | 35.0/39.3 | 60.3/65.5 | 63.4/47.1 |
| F wave latency (ms) | 25.7/25.2 | NR/NR | NR/27.9 | NR/NR | 33.9/32.6 | NR/NR |
| Conduction block | No | No | No | No | No | Yes |
| Temporal dispersion | No | No | Yes | Yes | No | No |
| **Tibial nerve** |  |  |  |  |  |  |
| Distal latency (ms) | 5.25/5.33 | 5.85/5.0 | 6.84/8.67 | 6.23/6.42 | 4.59/4.53 | 4.68/4.03 |
| CMAP amplitude (mv) | 1.02/1.2 | 3.5/2.6 | 1.0/0.67 | 0.42/1.32 | 6.5/6.0 | 4.0/6.4 |
| F wave latency (ms) | 54.0/54.1 | NR/NR | NR/NR | NR/NR | 62.2/64.7 | NR/57.7 |
| Conduction block | No | No | No | No | No | Yes |
| Temporal dispersion | No | No | Yes | Yes | No | No |
| **Peroneal nerve** |  |  |  |  |  |  |
| Distal latency (ms) | 7.71/7.51 | 11.9/5.07 | 11.6/14.3 | 8.65/4.31 | 4.72/4.75 | 3.91/4.7 |
| CMAP amplitude (mv) | 0.98/0.34 | 1.16/0.94 | 0.77/0.28 | 1.14/1.92 | 2.8/2.9 | 2.6/1.62 |
| Conduction velocity (m/s) | 35.1/35.5 | 50.7/31.1 | 21.6/25.6 | 39.3/33.0 | 45.3/45.4 | 50.9/23.3 |
| F wave latency (ms) | NR/NR | NR/NR | NR/NR | NR/NR | NR/60.9 | NR/NR |
| Conduction block | No | No | No | No | No | Yes |
| Temporal dispersion | No | No | Yes | Yes | No | No |
| **Sensory nerve conduction** |  |  |  |  |  |  |
| **Median nerve** |  |  |  |  |  |  |
| SNAP amplitude (μv) | 32.7/21.5 | NR/NR | NR/NR | NR/NR | 12.7/12.5 | 4.7/NR |
| Conduction velocity (m/s) | 50.5/49.5 | NR/NR | NR/NR | NR/NR | 48.8/49.0 | 48.1/NR |
| **Ulnar nerve** |  |  |  |  |  |  |
| SNAP amplitude (μv) | 10.9/7.2 | 2.2/NR | NR/NR | NR/NR | 7.6/7.3 | NR/NR |
| Conduction velocity (m/s) | 50.7/51.9 | 48.7/NR | NR/NR | NR/NR | 50.7/48.5 | NR/NR |
| **Tibial nerve** |  |  |  |  |  |  |
| SNAP amplitude (μv) | 0.7/0.74 | NR/NR | NR/NR | NR/NR | 0.78/0.55 | NR/NR |
| Conduction velocity (m/s) | 42.5/39.1 | NR/NR | NR/NR | NR/NR | 37.4/39.8 | NR/NR |
| **Sural nerve** |  |  |  |  |  |  |
| SNAP amplitude (μv) | 9.2/10.5 | 10.3/6.8 | NR/NR | 6.9/9.2 | 6.9/6.9 | 1.58/1.78 |
| Conduction velocity (m/s) | 56.6/56.1 | 49.8/53.5 | NR/NR | 57.6/63.8 | 58.5/52.4 | 53.7/51.2 |

**Supplementary Table S2 (continued)** The electrophysiological records of COVID-GBS patients.

| Patient | 7 | 8 | 9 | 10 | 11 | 12 | 13 |
| --- | --- | --- | --- | --- | --- | --- | --- |
| **Motor nerve conduction** |  |  |  |  |  |  |  |
| **Median nerve** |  |  |  |  |  |  |  |
| Distal latency (ms) | 17.5/20.8 | 12.2/NA | 3.91/3.78 | 2.84/3.11 | 7.32/8.96 | 2.86/3.09 | 6.55/7.96 |
| CMAP amplitude (mv) | 0.58/2.0 | 1.61/NA | 8.9/8.2 | 7.2/6.4 | 3.8/2.3 | 10.0/10.4 | 2.2/3.8 |
| Conduction velocity (m/s) | 37.7/35.1 | 31.9/NA | 70.8/66.1 | 53.7/54.7 | 46.0/41.2 | 57.3/57.3 | 45.5/41.2 |
| F wave latency (ms) | NR/36.6 | NR/NR | 22.7/23.8 | 31.7/30.2 | 29.7/NR | 20.5/21.2 | NR/33.0 |
| Conduction block | No | No | No | No | No | No | No |
| Temporal dispersion | No | No | No | No | No | No | Yes |
| **Ulnar nerve** |  |  |  |  |  |  |  |
| Distal latency (ms) | 4.64/5.41 | 8.67/8.42 | 3.09/3.08 | 2.34/2.74 | 4.93/7.08 | 1.89/2.09 | 4.71/4.42 |
| CMAP amplitude (mv) | 1.46/1.91 | 2.6/3.4 | 8.3/9.8 | 9.4/8.2 | 3.6/3.3 | 10.5/10.4 | 2.1/3.0 |
| Conduction velocity (m/s) | 47.2/49.1 | 37.1/40.1 | 53.9/61.9 | 60.9/61.6 | 45.6/44.7 | 62.5/64.5 | 49.4/49.3 |
| F wave latency (ms) | 32.7/27.1 | NR/NR | 23.4/24.7 | 33.9/32.5 | 32.3/31.8 | 21.3/20.9 | 31.8/32.9 |
| Conduction block | No | No | No | No | No | No | No |
| Temporal dispersion | No | No | No | No | No | No | Yes |
| **Tibial nerve** |  |  |  |  |  |  |  |
| Distal latency (ms) | 9.59/9.58 | 14.4/11.0 | 4.79/4.06 | 3.43/3.35 | 7.27/7.22 | 2.99/3.17 | 10.4/8.86 |
| CMAP amplitude (mv) | 0.33/0.51 | 0.26/0.29 | 8.3/7.5 | 10.5/9.6 | 1.64/1.5 | 13.5/16.3 | 2.0/3.2 |
| F wave latency (ms) | NR/NR | NR/NR | 43.1/41.6 | 51.5/52.5 | NR/NR | 40.2/40.4 | 62.1/62.4 |
| Conduction block | No | No | No | No | No | No | No |
| Temporal dispersion | Yes | Yes | No | No | No | No | Yes |
| **Peroneal nerve** |  |  |  |  |  |  |  |
| Distal latency (ms) | 8.18/9.97 | 13.4/11.3 | 4.46/4.64 | 3.83/3.81 | 5.68/6.65 | 3.3/3.3 | 9.55/12.0 |
| CMAP amplitude (mv) | 2.0/1.18 | 0.14/0.20 | 1.6/1.43 | 3.3/2.4 | 2.3/0.94 | 4.7/4.4 | 2.5/1.92 |
| Conduction velocity (m/s) | 31.9/32.5 | 19.7/22.6 | 46.2/46.0 | 45.8/46.3 | 34.0/33.9 | 54.2/52.0 | 34.2/34.4 |
| F wave latency (ms) | NR/NR | NR/NR | 46.0/44.3 | 59.5/61.9 | NR/NR | 41.6/42.9 | NR/NR |
| Conduction block | No | No | No | No | No | No | No |
| Temporal dispersion | No | Yes | No | No | No | No | Yes |
| **Sensory nerve conduction** |  |  |  |  |  |  |  |
| **Median nerve** |  |  |  |  |  |  |  |
| SNAP amplitude (μv) | NR/NR | NR/NR | 22.7/32.4 | 32.4/24.5 | 1.13/1.41 | 37.9/38.3 | NR/NR |
| Conduction velocity (m/s) | NR/NR | NR/NR | 48.5/50.5 | 52.6/49.0 | 46.5/47.6 | 58.4/57.7 | NR/NR |
| **Ulnar nerve** |  |  |  |  |  |  |  |
| SNAP amplitude (μv) | NR/NR | NR/NR | 13.4/13.5 | 13.8/10.8 | NR/NR | 17.3/14.8 | NR/NR |
| Conduction velocity (m/s) | NR/NR | NR/NR | 52.0/47.9 | 54.5/54.2 | NR/NR | 58.1/59.5 | NR/NR |
| **Tibial nerve** |  |  |  |  |  |  |  |
| SNAP amplitude (μv) | NR/NR | NR/NR | 1.4/2.3 | 0.46/0.48 | NR/NR | 4.4/3.7 | NR/NR |
| Conduction velocity (m/s) | NR/NR | NR/NR | 39.4/39.4 | 35.9/37.2 | NR/NR | 43.7/45.2 | NR/NR |
| **Sural nerve** |  |  |  |  |  |  |  |
| SNAP amplitude (μv) | NR/NR | NR/NR | 3.8/4.8 | 8.0/8.4 | NR/NR | 11.3/9.4 | 6.7/7.1 |
| Conduction velocity (m/s) | NR/NR | NR/NR | 48.5/56.8 | 53.0/57.1 | NR/NR | 58.6/59.1 | 57.6/57.6 |

Abbreviations: NR = not responsible; NA = not available.

Note: Motor and sensory nerve conduction results are presented as Left value / Right value.
